# Peroxymonosulfate-Activation-Induced Phase Transition of Mn_3_O_4_ Nanospheres on Nickel Foam with Enhanced Catalytic Performance

**DOI:** 10.3390/molecules28114312

**Published:** 2023-05-24

**Authors:** Cuiyin Liu, Ziyan Wang, Yanfeng Chen, Xinjuan Zeng, Hangyu Long, Haibo Rong, Hongtao Zou, Jinpeng Ding, Jingling Li

**Affiliations:** 1 School of Materials Science and Hydrogen Energy, Foshan University, Foshan 528000, China; liu_cuiyin@163.com (C.L.);; 2 Guangdong Key Laboratory for Hydrogen Energy Technologies, Foshan 528000, China; 3 Guangdong Provincial Key Laboratory of Distributed Energy Systems, School of Chemical Engineering and Energy Technology, Dongguan University of Technology, Dongguan 523808, China; 4 School of Light Industry and Materials, Guangdong Polytechnic, Foshan 528041, China

**Keywords:** Mn_3_O_4_, peroxymonosulfate activation, phase transition, morphological change

## Abstract

The transformations of physicochemical properties on manganese oxides during peroxymonosulfate (PMS) activation are vital factors to be concerned. In this work, Mn_3_O_4_ nanospheres homogeneously loaded on nickel foam are prepared, and the catalytic performance for PMS activation is evaluated by degrading a target pollutant, Acid Orange 7, in aqueous solution. The factors including catalyst loading, nickel foam substrate, and degradation conditions have been investigated. Additionally, the transformations of crystal structure, surface chemistry, and morphology on the catalyst have been explored. The results show that sufficient catalyst loading and the support of nickel foam play significant roles in the catalytic reactivity. A phase transition from spinel Mn_3_O_4_ to layered birnessite, accompanied by a morphological change from nanospheres to laminae, is clarified during the PMS activation. The electrochemical analysis reveals that more favorable electronic transfer and ionic diffusion occur after the phase transition so as to enhance catalytic performance. The generated SO_4_^•−^ and •OH radicals through redox reactions of Mn are demonstrated to account for the pollutant degradation. This work will provide new understandings of PMS activation by manganese oxides with high catalytic activity and reusability.

## 1. Introduction

Advanced oxidation processes (AOPs) with free radicals have received much attention for the various applications in environmental remediation, including degradation and mineralization of organic molecules [1], detoxification [2], and disintegration of sludge [3]. The sulfate radical (SO_4_^•−^) provided tremendous potential as an advanced alternative to hydroxyl radical (•OH) due to its advantages including higher standard redox potential ($E_{{\mathrm{SO}}_{4}^{•-}}$ = 2.5–3.1 V, $E_{•\mathrm{OH}}$ = 1.8–2.7 V, vs. NHE) [4,5], better practicability under a wide pH range (pH = 2–8) [6,7], and longer lifespan (30–40 μs for SO_4_^•−^ vs. 20 ns for •OH) [8,9]. It has been reported that SO_4_^•−^ could be generated by the activation of peroxydisulfate (PDS) or peroxymonosulfate (PMS) with heating [10], UV irradiation [11], and ultrasound [12]. These physical activation methods require persistent energy inputs, resulting in higher costs for practical applications. Homogeneous transition-metal ions in solution, such as Co^2+^ [13], Fe^2+^ [14], and Cu^2+^ [15], can generate SO_4_^•−^ through the change in valence state but without extraneous energy inputs. However, the homogeneous transition-metal ions are difficult to reclaim, and may cause secondary pollution. To overcome these problems, various heterogeneous catalysts, particularly transition-metal oxides, have been extensively investigated in PMS and PDS activation. 

Among all kinds of transition-metal oxides, manganese oxides are promising catalysts for the effective activation of PMS due to their advantages of diverse valency, high natural abundance, and environmental benignity [16,17]. The reactivity of manganese oxides can be influenced by different structural and surface physicochemical properties. Manganese oxides with different chemical states, including MnO, MnO_2_, Mn_2_O_3,_ and Mn_3_O_4_, displayed catalytic diversity on PMS activation [18]. In addition, the reactivity of different crystallographic MnO_2_ had a high correlation with the surface Mn valence state, and other properties, such as surface area, conductivity, or surface adsorbed oxygen, were secondary factors [19]. Therefore, it is essential to explore the diversify the oxidation states of Mn ions in manganese oxides. Thermodynamically stable hausmannite Mn_3_O_4_ composed of Mn(II) in tetrahedral sites and Mn(III) in octahedral sites has been reported as a promising catalyst alternative to Co_3_O_4_ and Fe_3_O_4_ for PMS-based AOPs [20]. However, Mn_3_O_4_ generally suffers from poor conductivity and serious agglomeration [21], which would hinder the electron transfer and exposure of active sites. 

To tackle with above issues, the construction of novel microstructures for Mn_3_O_4_ is one of the strategies, such as hierarchical nanostructures [22] and yolk–shell microparticles [23]. In addition, the employment of matrixes (g-C_3_N_4_ [24] and reduced graphene oxide [25]) can effectively improve Mn_3_O_4_ spatial configuration. Previous studies have demonstrated nickel foam as a supporter for the uniform distribution of nano/micro materials, such as Co(OH)_2_ nanosheets [26], CuCoNi oxide nanowires [27], and Cu/CoS nano needles [28], which deliver high catalytic performance. Thus, the employment of nickel foam for the support of Mn_3_O_4_ is worthy of study. Moreover, the chemical phases of Mn_3_O_4_ are difficult to retain after PMS activation, affecting the reusability [29,30]. It has been reported that the chemical valence states of Mn_3_O_4_ are variational during PMS activation [29]. In addition to the valence states, the accompanying changes in crystal structure and morphology have not been considered in the reported literatures. Thus, detailed investigations of the transformations of physicochemical properties on Mn_3_O_4_ during PMS activation are vital for approaching high catalytic stability.

Herein, Mn_3_O_4_ nanospheres homogeneously loaded on nickel foam were constructed for PMS activation to degrade Acid Orange 7 (AO7, a typical dye). The influence of catalyst loading, nickel foam substrate, and some conditions, including PMS concentration, pH value, and reaction temperature, were investigated in detail. The cyclic performance accompanied by the changes in the microstructure and surface chemistry of Mn_3_O_4_ during PMS activation was explored. A solid-phase transition from spinel Mn_3_O_4_ to birnessite induced by PMS activation was clarified. Improved electronic transfer and ionic diffusion of the catalyst after repeated use were revealed, which were responsible for the promotion of the degradation rate. A mechanism involved in the solid-phase transition of Mn_3_O_4_ and the generation of SO_4_^•−^ and •OH radicals was proposed.

## 2. Results and Discussion

### 2.1. Characterization of the As-Prepared Mn_3_O_4_ Sample

**Figure 1:**
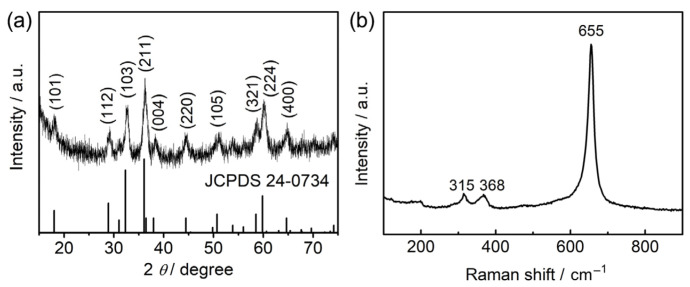
As-prepared Mn_3_O_4_ sample: (**a**) XRD pattern; (**b**) Raman spectrum.

The XRD pattern of the as-prepared Mn_3_O_4_ sample scraped from nickel foam is shown in Figure 1a. All the diffraction peaks are well indexed to hausmannite Mn_3_O_4_ (JCPDS 24-0734), the most prominent of which at 2θ values of 32.7° and 36.2° can be attributed to the (103) and (211) planes of the tetragonal spinel structure. Raman and FT-IR measurements were carried out to analyze the phase purity of the sample. As shown in Figure 1b, the strong Raman band located at 655 cm^−1^ is characteristic of hausmannite Mn_3_O_4_, which arose due to the breathing mode of Mn-O vibration from Mn^2+^ ions on the tetrahedral site [31]. Two main absorption bands located at 630 and 511 cm^−1^ are observed from the FT-IR spectrum shown in Figure S1. This result agrees with the previously reported Mn_3_O_4_, which is attributed to Mn-O stretching vibrations on octahedral and tetrahedral sites [22]. The broad absorption band at around 3400 cm^−1^ could be assigned to the stretching vibration of the adsorbed hydroxyl group. These spectroscopic results further confirm the Mn_3_O_4_ phase.

**Figure 2:**
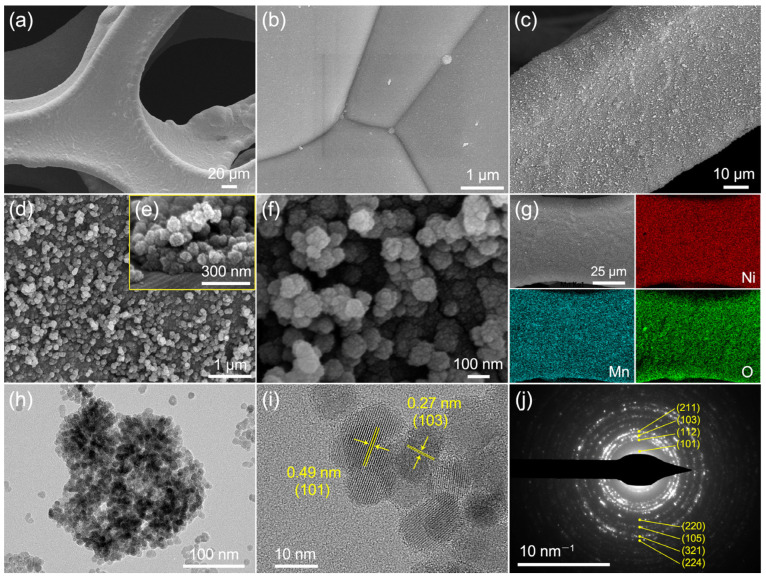
(**a**,**b**) SEM images of the pristine nickel foam. As-prepared Mn_3_O_4_ sample: (**c**–**f**) SEM images; (**g**) EDS element mappings; (**h**,**i**) TEM images; (**j**) SAED pattern.

The SEM images of the pristine nickel foam and the as-prepared Mn_3_O_4_ sample are present. Figure 2a,b display the 3D porous structure of the nickel foam and its rather smooth surface. After the hydrothermal reaction, the evenly coated Mn_3_O_4_ nanospheres roughen the nickel foam surface (Figure 2c,d). The nanospheres stacked on top of each other forming an irregular thickness (Figure 2e). The enlarged SEM image in Figure 2f reveals the irregularly spherical shape of Mn_3_O_4_ nanospheres with a diameter of about 100–200 nm. The EDS results show the homogeneous distribution of Ni, Mn, and O elements on the sample (Figure 2g). The TEM image exhibits Mn_3_O_4_ nanospheres formed by the aggregation of small nanoparticles (Figure 2h). The nanoparticles are approximately 12 nm in size. The high-resolution TEM (HRTEM) image in Figure 2i displays lattice fringes with interplanar distances of 0.49 and 0.27 nm, which correspond to the crystal planes of (101) and (103) of hausmannite Mn_3_O_4_, respectively. The selected area electron diffraction (SAED) pattern obtained from Figure 2j demonstrates a polycrystalline feature and can be well indexed to hausmannite Mn_3_O_4_.

### 2.2. Degradation Performances in Different Systems

#### 2.2.1. Effect of Catalyst Loading

**Figure 3:**
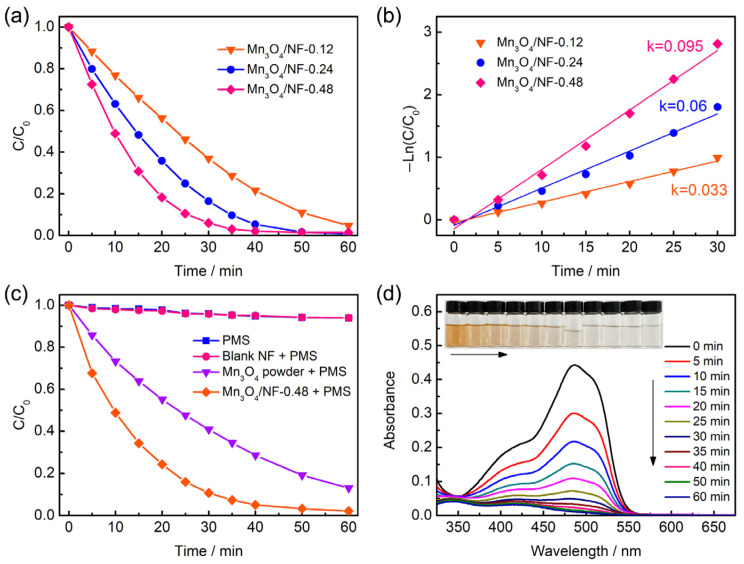
AO7 degradation efficiency (**a**) and kinetic curves (**b**) of Mn_3_O_4_/NF-0.12, Mn_3_O_4_/NF-0.24, and Mn_3_O_4_/NF-0.48. (**c**) AO7 degradation efficiency of Mn_3_O_4_/NF-0.48 under different systems. (**d**) UV-vis spectra of AO7 solution under given time intervals in the presences of Mn_3_O_4_/NF-0.48 and PMS. Conditions: [AO7]_0_ = 20 mg/L, [oxone]_0_ = 1 mM.

The samples with different catalyst loading were prepared using different dosages of manganese salt (Mn(CH_3_COO)_2_∙4H_2_O). The prepared samples were denoted as Mn_3_O_4_/NF-M (M means the dosage of manganese salt). The mass loadings of Mn_3_O_4_/NF-0.12, Mn_3_O_4_/NF-0.24, and Mn_3_O_4_/NF-0.48 are about 0.3, 0.6, and 0.9 mg cm^−2^, respectively. The SEM images of Mn_3_O_4_/NF-0.24 and Mn_3_O_4_/NF-0.12 are present in Figure S2. It can be observed that the Mn_3_O_4_ nanospheres aggregated by nanoparticles were uniformly distributed on the nickel foam surface. In contrast with the morphology of Mn_3_O_4_/NF-0.48, the Mn_3_O_4_ nanospheres exhibited decreasingly smaller dimensions with lower dosages of manganese salt. Figure 3a shows the AO7 degradation of different samples under the same conditions. The Mn_3_O_4_/NF-0.48 presented a better PMS activation performance than Mn_3_O_4_/NF-0.24 and Mn_3_O_4_/NF-0.12. A degradation efficiency of 94% could be achieved for Mn_3_O_4_/NF-0.48 within 30 min, which was 83.5% and 63% for Mn_3_O_4_/NF-0.24 and Mn_3_O_4_/NF-0.12, respectively. The results of AO7 degradation with the Mn_3_O_4_/NF system could be fitted to pseudo-first-order kinetics, as shown in Figure 3b. The degradation rate constant (k) of Mn_3_O_4_/NF-0.48 was estimated to be 0.095 min^−1^, higher than those of Mn_3_O_4_/NF-0.24 (0.060 min^−1^) and Mn_3_O_4_/NF-0.12 (0.033 min^−1^). The better degradation performance of Mn3O4/NF-0.48 could be attributed to its raised catalyst loading.

#### 2.2.2. Effect of Nickel Foam

The effect of nickel foam was evaluated by the comparison of the performance using blank nickel foam, Mn_3_O_4_ powder, and Mn_3_O_4_/NF-0.48. Figure 3c displays AO7 degradation under different systems. A low degradation efficiency of about 6% was evaluated after 60 min with the presence of PMS or both PMS and blank nickel foam, suggesting that the blank nickel foam shows negligible effectiveness on the removal of AO7. The degradation efficiency of Mn_3_O_4_ powder with PMS (87%) is lower than that of Mn_3_O_4_/NF-0.48 with PMS (98%) after 60 min. Furthermore, no adsorption of AO7 on Mn_3_O_4_/NF-0.48 was detected (Figure S3) within 30 min, confirming the contribution of nickel foam to the catalytic activity of Mn_3_O_4_. Figure 3d shows the corresponding UV-vis spectra of the reaction system as a function of degradation time. The consecutive reduction in the absorbance at around 484 nm proved the destruction of azo chromophore.

#### 2.2.3. Effects of Degradation Conditions

**Figure 4:**
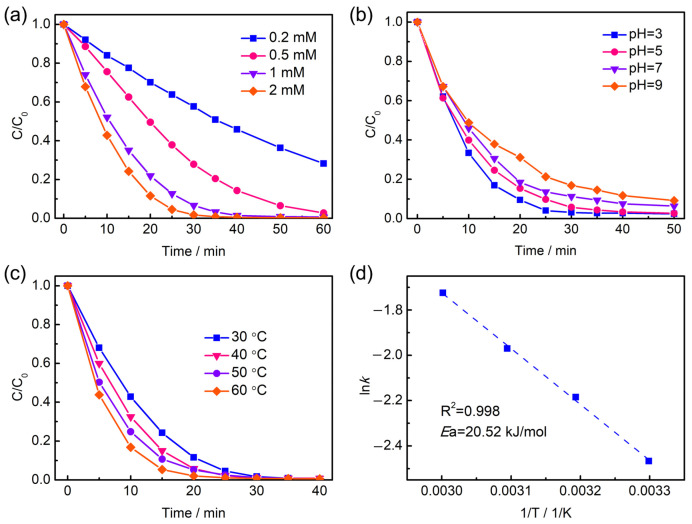
The influences of PMS concentration (**a**), initial pH value (**b**), and reaction temperature (**c**) on AO7 degradation with Mn_3_O_4_/NF-0.48. (**d**) The corresponding Arrhenius curve at different reaction temperatures. Conditions: (**a**) [AO7]_0_ = 20 mg/L; (**b**,**c**) [AO7]_0_ = 20 mg/L, [oxone]_0_ = 1 mM.

The effects of PMS concentration, initial pH value, and reaction temperature have been investigated in the Mn_3_O_4_/NF-0.48 system. As shown in Figure 4a, PMS concentration promotes the degradation efficiency significantly in the range of 0.2 to 1 mM oxone, which could be ascribed to the involvement of more reactive species activated from PMS. Once the concentration of PMS is higher than 1 mM, the positive effect on degradation tends to be slight. In the current reaction system, the PMS dosage is in plenty relative to the catalyst loaded on nickel foam. In consideration of the degradation efficiency and operation cost, 1 mM of PMS concentration can be more adequate for AO7 degradation. 

The influence of the initial pH value on the degradation of AO7 was examined. As shown in Figure 4b, a weakening trend of degradation performance with an increasing initial pH value can be observed. It is reported that manganese oxides are generally negatively charged under neutral and basic conditions [30,32]. The electrostatic repulsion between the catalyst and PMS anion or anionic dye AO7 might be one of the reasons. In addition, the deprotonation of HSO_5_^−^ into SO_4_^2−^ and O_2_ under basic conditions would cause invalid PMS consumption [33]. Nonetheless, the system of Mn_3_O_4_/NF-0.48 with PMS shows efficiency in the pH range from 3 to 9, since the degradation efficiencies remain above 90%.

The effect of the reaction temperature on AO7 degradation is displayed in Figure 4c, showing an accelerated degradation at higher temperature. The degradation rate constant at 30, 40, 50, and 60 °C can be deduced to be 0.085, 0.112, 0.139, and 0.178 min^−1^. The relationship between the rate constants and reaction temperatures obeys the Arrhenius equation closely as shown in Figure 4d. The activation energy (*E*_a_) is estimated to be 20.52 kJ/mol, which is at a lower level than those of AO7 degradation over some reported catalysts, for example, Mn_3_O_4_-rGO (49.5 kJ/mol) [25], MnFe_2_O_4_ (31.7 kJ/mol) [34], Co/Bi_25_FeO_40_ (51.3 kJ/mol) [35], and Co_3_O_4_/N-doped graphene (41.6 kJ/mol) [36]. It suggests that the Mn_3_O_4_/NF exhibits a low chemical reaction energy barrier as a promising catalyst for PMS activation. The influence of the stirring rate on AO7 degradation was explored. As shown in Figure S4, a degradation rate constant (k) of 0.094 min^−1^ could be achieved under the stirring rate of 200 rpm, which was 0.024 and 0.04 min^−1^ under the stirring rates of 50 and 400 rpm, respectively. Thus, a stirring rate of 200 rpm is suitable for mass transfer.

### 2.3. Catalyst Reusability and Structural Change

**Figure 5:**
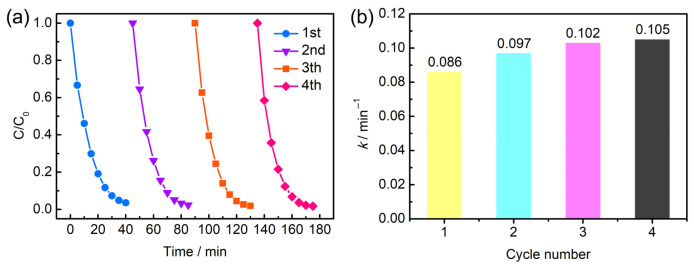
(**a**) Recycling study of AO7 degradation. (**b**) Pseudo-first-order kinetic constant (*k*, min^−1^) versus cycle number.

Figure 5a displays the recycling performance of AO7 degradation with Mn_3_O_4_/NF-0.48. As the results show, the catalyst exhibited progressively enhanced degradation performance within the four repeated cycles. The AO7 degradation rate escalated from 0.086 to 0.105 min^−1^ after four runs (Figure 5b). These results are noteworthy as the heterogeneous catalysts usually suffer from the problem of stability. 

**Figure 6:**
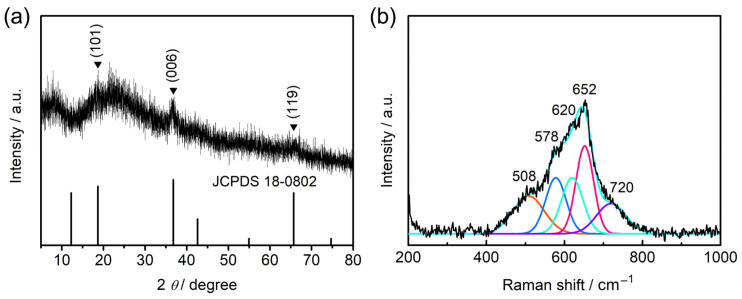
Catalyst sample after four cycles of PMS activation on AO7 degradation: (**a**) XRD pattern; (**b**) Raman spectrum.

The catalyst after four cycles of repetitive usages was further investigated to make clear the reason for enhanced degradation performance. The XRD pattern in Figure 6a demonstrates a set of characteristic peaks that can be indexed to the standard contour of birnessite MnO_2_ (JCPDS no. 18-0802). The diffraction peaks at 2θ values of 18.7°, 36.8°, and 65.7° can be attributed to the (101), (006), and (119) planes of the layered structure. These weak diffraction peaks indicate the low crystallinity of the catalyst. The broad peak at the range of 20° to 30° may be ascribed to the adsorbed organic intermediates on the catalyst surface [37]. Figure 6b displays deconvoluted Raman bands at 508, 578, 652, and 720 cm^−1^, which could be assigned to the vibrational features of birnessite manganese oxide [38]. Additionally, the band at 620 cm^−1^ may be raised due to the presence of MnOOH on the catalyst surface [39]. These results attest to a phase transition from spinel Mn_3_O_4_ to birnessite manganese oxide after PMS activation on AO7 degradation.

**Figure 7:**
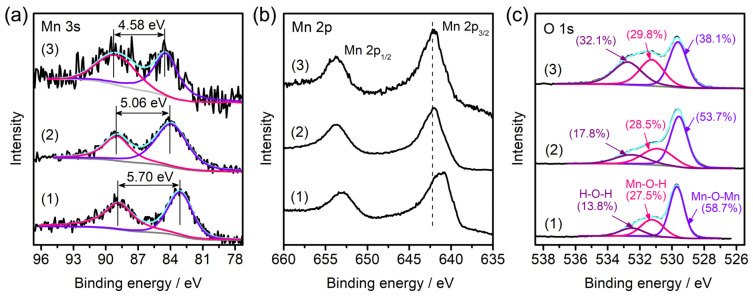
Comparisons of Mn 3s (**a**), Mn 2p (**b**), and O 1s (**c**) core-level XPS spectra of Mn_3_O_4_/NF-0.48 (1) and the catalyst samples after one cycle (2) and four cycles (3) of AO7 degradation.

XPS was employed to analyze the evolution of the surface chemistry on the catalyst sample. From the Mn 3s core-level XPS spectra (Figure 7a), the energy separation of the two peaks was measured to be 5.70, 5.06, and 4.58 eV for pristine Mn_3_O_4_/NF-0.48 and the samples after one cycle and four cycles, respectively. This value has been proved to be linearly corelated to the mean valence state of Mn [40], which then can be estimated to be +2.50, +3.23, and +3.77 for the three samples, respectively. This verifies the increasing trend of the mixed Mn valence states during the repeated PMS activation on AO7 degradation. The shifts of the broad Mn 2p_3/2_ peak toward higher binding energy of the samples after one cycle and four cycles (Figure 7b) further confirm the oxidation of Mn. The O 1s spectra (Figure 7c) are deconvoluted into three bands which, respectively, correspond to the Mn-O-Mn bond (529.6 ± 0.1 eV) for anhydrous Mn oxide, Mn-O-H bond (531.1 ± 0.2 eV) for Mn hydroxide, and H-O-H bond (532.5 ± 0.2 eV) for structure water. Increasing content of the surface hydroxyl group and structure water were detected during the repeated cycles. In particular, more than twice the content of the structure water on the surface was obtained after four cycles of degradation.

**Figure 8:**
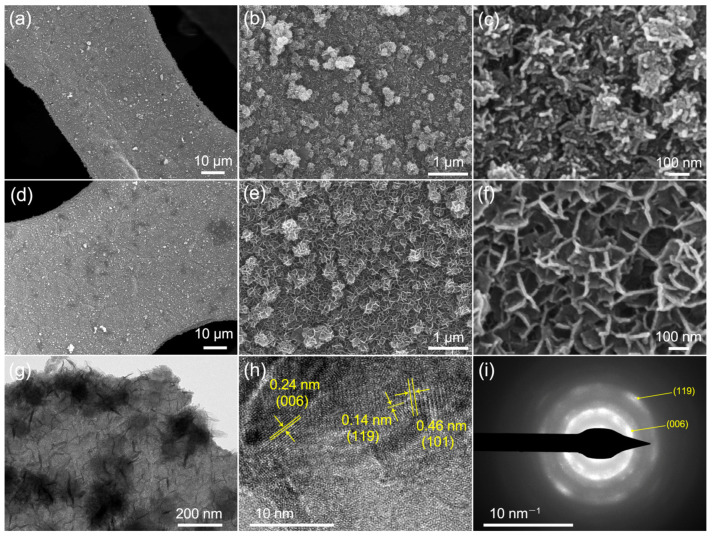
SEM images of the catalyst sample after one cycle (**a**–**c**) and four cycles (**d**–**f**) of AO7 degradation. Sample after four cycles: (**g**,**h**) TEM images; (**i**) SAED pattern.

The surface morphologies of the catalyst samples collected after the first and fourth cycles were observed using SEM. As shown in Figure 8a–c, the pristine Mn_3_O_4_ nanospheres in situ grew into a lamella structure. With an increasing cycle number, the lamellae exhibited a larger lateral diameter, and interconnected with each other assembling porous architecture (Figure 8d–f). The corresponding TEM image (Figure 8g) displays the interconnected lamellae, which are 5–20 nm in thickness and 50–200 nm in lateral size. The HRTEM image (Figure 8h) shows the typical lattice fringes with spacings of 0.14, 0.24, and 0.46 nm, which correspond to the (119), (006), and (101) planes of birnessite MnO_2_, respectively. The SAED pattern (Figure 8i) further confirms the poor crystallinity of the birnessite structure, which agrees well with the XRD result.

**Figure 9:**
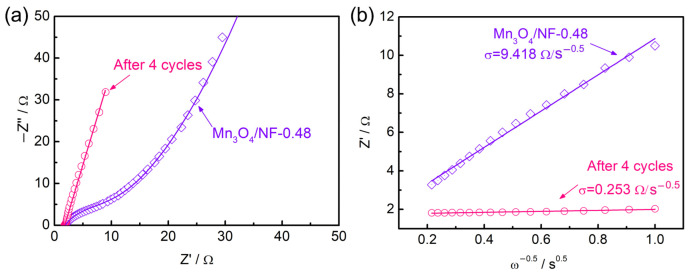
(**a**) Nyquist plots of the Mn_3_O_4_/NF-0.48 and the sample after four cycles of AO7 degradation. (**b**) Z’ versus the reciprocal of the square root of frequency (ω^−0.5^) in the intermediate frequency range.

Based on the abovementioned results, it can be seen that Mn_3_O_4_ catalyst samples suffer a chemical oxidation process after AO7 degradation with PMS activation, leading to a solid-phase transition from spinel to birnessite and morphological evolution from nanospheres to a lamella structure. The enhanced degradation performance of the catalyst with repeated use is most probably associated with the phase and morphology changes. The properties of electron transport and ion diffusion resistivity of the catalysts are analyzed using electrochemical impedance spectroscopy (EIS). Nyquist plots and the fitting lines of the Mn_3_O_4_/NF-0.48 and the sample after four cycles of AO7 degradation are compared in Figure 9a. The approximate semicircle at high frequency represents a charge-transfer-controlled process. The series resistance (R_s_) and charge-transfer resistance (R_ct_) are fitted to be 1.906 Ω and 9.858 Ω for Mn_3_O_4_/NF-0.48, while they change to be 1.797 Ω and 0.01 Ω after four cycles. The phase transition introduces minimal change to the intrinsic resistance of the catalyst but a great extent of reduction on the R_ct_. This indicates better efficient electron transfer at the interface, which could promote the reaction between the catalyst and PMS. The straight line at low frequency represents an ion-diffusion-controlled process. The higher slop of the line implies a more favorable ionic diffusion of the catalyst after four cycles. The real part of impedance (Z’) is plotted versus the reciprocal of the square root of frequency (ω^−0.5^) in the intermediate frequency range, which can derive the ion diffusion resistance (σ) through the slope of linear fitting (Figure 9b). The σ value is found to decrease considerably from 9.418 Ω/s^−0.5^ to 0.253 Ω/s^−0.5^ after four cycles. The decrease in σ may be ascribed to the lamella structure, which offers open channels for facile ion diffusion [22]. This allows more active surface sites for the adsorption and reactions of the reactants, improving the catalyst activity.

### 2.4. Identification of Radicals

**Figure 10:**
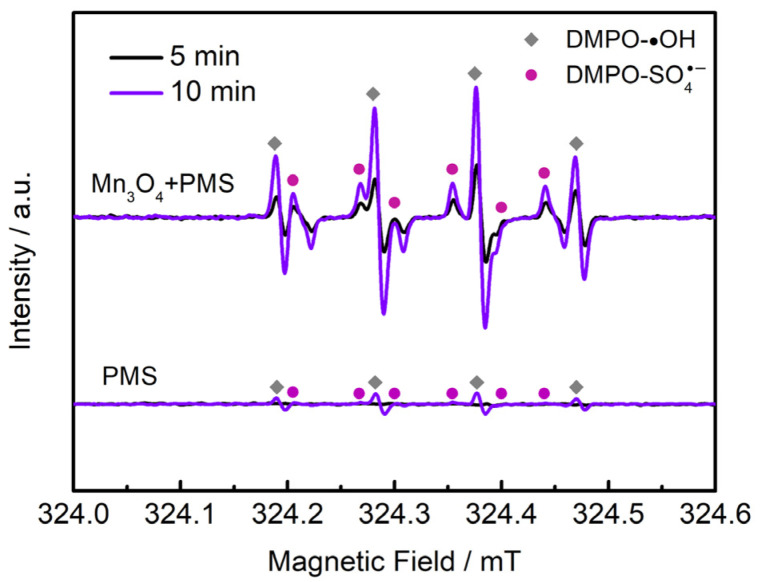
EPR spectra of Mn_3_O_4_/NF-0.48/PMS system captured by DMPO.

It has been reported that the organic pollutant degradation with PMS usually follows two different mechanisms, including radical and nonradical pathways. EPR was employed to identify the active species for catalysis. The radical signals captured by DMPO are shown in Figure 10. Compared with the weak signals without catalyst addition, high intensities of DMPO-•OH and DMPO- SO_4_^•−^ signals could be observed in the Mn_3_O_4_/NF-0.48/PMS system. Both signals exhibit enhanced intensities with the increase in reaction time from 5 min to 10 min. It suggests that both •OH and SO_4_^•−^ could be derived via catalysis during PMS activation and would be accumulated within the reaction time. In addition, the nonradical ^1^O_2_ signal captured using TEMP is present in Figure S5. The TEMP-^1^O_2_ signal in the Mn_3_O_4_/NF-0.48/PMS system showed roughly the same intensity as that of the raw PMS system without catalyst addition. It indicates that nonradical ^1^O_2_ formed through spontaneous PMS decomposition, which presented little contribution to the AO7 degradation based on the above results under the raw PMS condition. Therefore, radical •OH and SO_4_^•−^ were the contributing species for the AO7 degradation in the Mn_3_O_4_/NF-0.48/PMS system.

### 2.5. The Mechanism of PMS Activation and Phase Transition

**Figure 11:**
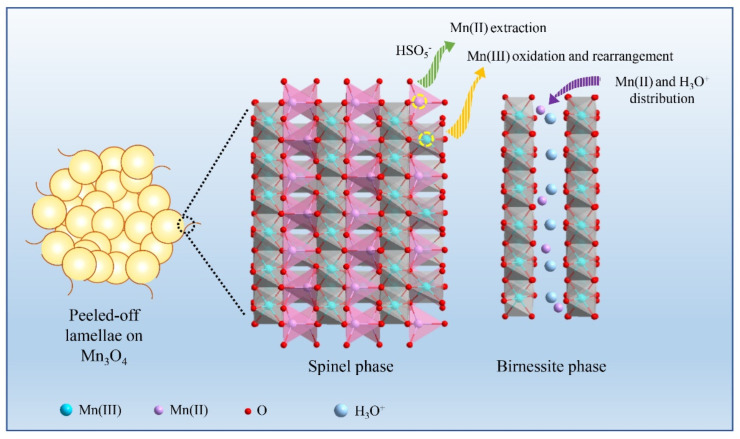
Scheme for the phase transition induced by PMS activation.

Based on above results, the phase transition induced by PMS activation was schematically proposed in Figure 11. Firstly, Mn(II) can produce SO_4_^•−^ by reacting with HSO_5_^−^ (Equation (1)). The oxidation reaction of Mn(II) may induce its extraction from tetrahedral sites, which is similar to the dissolution of Mn(II) form Mn_3_O_4_ matrix under electrochemical oxidation conditions in aqueous solution [41,42]. Meanwhile, Mn(III) at the octahedral sites can be oxidized to Mn(IV), while one fourth of octahedron Mn cations migrate to the (101) plane of spinel, leading to the in situ formation of a layered structure [43]. Additionally, Mn at the higher valence states can then be reduced by HSO_5_^−^ to generate SO_4_^•−^ (Equations (2) and (3)). The generated SO_4_^•−^ can be readily converted to •OH via the oxidation of water (Equation (4)). Based on the surface chemistry of the catalyst after repeated cycles, the extracted Mn(II) and free H_3_O^+^ may fill into the MnO_6_ layer gap to recover the charge balance after the ion extraction and stabilize the structure [44]. These atomic movements during the solid-phase transition produce a large strain between the phase edges, which could cause the grown birnessite lamella to peel off from the Mn_3_O_4_ surface [45], leading to morphological change.

(1) $${{\mathrm{HSO}}_{5}}^{-}+\equiv \mathrm{Mn}(\mathrm{II})\;\to \;{{\mathrm{SO}}_{4}}^{\text{•}-}+{\mathrm{OH}}^{-}+\equiv \mathrm{Mn}(\mathrm{III})$$

(2) $${{\mathrm{HSO}}_{5}}^{-}+\equiv \mathrm{Mn}(\mathrm{III}/\mathrm{IV})\;\to \;{{\mathrm{SO}}_{5}}^{\text{•}-}+H^{+}+\equiv \mathrm{Mn}(\mathrm{II}/\mathrm{III})$$

(3) $$2{{\mathrm{SO}}_{5}}^{\text{•}-}\;\to \;2{{\mathrm{SO}}_{4}}^{\text{•}-}+O_{2}$$

(4) $${{\mathrm{SO}}_{4}}^{\text{•}-}+H_{2}O\;\to \;{{\mathrm{HSO}}_{4}}^{-}+{}^{\text{•}}\mathrm{OH}$$

## 3. Materials and Methods

### 3.1. Synthesis of Mn_3_O_4_ Nanospheres on Nickel Foam

Mn_3_O_4_ nanospheres were fabricated on nickel foam via a facile hydrothermal route, which was modified based on a reported recipe [46]. In brief, a precursor solution was firstly prepared through the dissolution of 0.48 g Mn(CH_3_COO)_2_∙4H_2_O into 3 g ethanol with 1 g deionized water under vigorous stirring for 15 min, followed by the addition of 13 mL ethylene glycol with continuous stirring for 30 min. A piece of pretreated nickel foam (~320 g m^−2^, 4 cm × 3.5 cm × 0.1 cm) was dipped into the above precursor solution. The pretreatment of nickel foam was conducted via drying after successive ultrasonic cleaning in acetone, ethanol, and deionized water, respectively. The solution and the nickel foam were statically aged for 3 days and then transferred into an autoclave for a hydrothermal reaction at 170 °C for 5 h. Finally, the nickel foam sample was removed and rinsed with deionized water and ethanol, and dried in air at 50 °C. For comparison, blank nickel foam and Mn_3_O_4_ powder were prepared using the same route but without the addition of manganese salt or nickel foam, respectively.

### 3.2. Characterization

The X-ray diffraction (XRD, SmartLab, Rigaku, Tokyo, Japan) was conducted using Cu-Kα radiation (*λ* = 1.5406 Å). Raman scattering was recorded using a LabRAM HR Evolution Raman spectrometer with a 532 nm laser (Horiba Jobin-Yvon, Villeneuve d’Ascq, France). Fourier transform infrared (FT-IR) was performed using a Fourier transform infrared spectrometer (Shimadzu IRAffinity-1S, Kyoto, Japan). The morphology was observed via field emission scanning electron microscopy (FESEM, Hitachi S4800, Kyoto, Japan) and field-emission transmission electron microscopy (TEM, FEI Tecnai TF20, Hillsboro, OR, USA). The elemental energy dispersive spectroscopy (EDS) analysis was conducted using an Oxford EDS detector. X-ray photoelectron spectroscopy (XPS) was performed using a monochromatic Al K*α* X-ray source with photon energy of 1486.6 eV (Thermo Scientific K-Alpha, ThermoFisher, Waltham, MA, USA). Electrochemical impedance spectroscopy (EIS) was performed on a CHI760E electrochemical workstation (Chenhua, China) with a Pt wire counter electrode and an Ag/AgCl (3 M KCl) reference electrode. An electron paramagnetic resonance spectrometer (EPR, JEOL JES FA200, Tokyo, Japan) was used to identify the radical species with DMPO and TEMP spin-trapping agents. 

### 3.3. Evaluation of Catalytic Activity

The catalytic activity was evaluated using the degradation of AO7 under a constant stirring of 200 rpm with the nickel foam sample at room temperature. In a typical procedure, the nickel foam sample was firstly immersed into 100 mL 20 mg L^−1^ of AO7 solution in a 250 mL beaker. After constant stirring for 30 min, 1 mmol/L oxone (2KHSO_5_•KHSO_4_•K_2_SO_4_) was added to initiate the reaction. At given time intervals, 2 mL solution samples were collected, followed by the addition of 2 mL ethanol for quenching of the reaction. The concentration of AO7 was detected using a UV-vis spectrophotometer (Shimadzu UV-1800, Japan) at 485 nm. For comparison, the catalytic activity of blank nickel foam and Mn_3_O_4_ powder was evaluated through the same route. The effect of pH was investigated using 1 M HCl or 1 M NaOH. The stability of the catalyst was evaluated through reutilization of the nickel foam sample after washing and drying within four successive degradation cycles.

## 4. Conclusions

In summary, Mn_3_O_4_ nanospheres homogeneously loaded on nickel foam are prepared using a simple hydrothermal route for PMS activation to degrade AO7. The Mn_3_O_4_/NF/PMS system displays favorable catalytic activity and an enhanced degradation rate with repeated usage. A transition from spinel nanospheres to birnessite laminae driven by PMS is revealed, which can lead to more favorable electronic transfer and ionic diffusion. The phase transition is proposed to be comprised of Mn extraction, rearrangement, and the insertion of cations between layers, accompanied by redox reactions of Mn to generate active radicals of SO_4_^•−^ and •OH. This work will provide new insights into PMS activation by manganese oxides.

## Data Availability

Data are available with requirements.

## Supplementary Materials

The following supporting information can be downloaded at https://www.mdpi.com/article/10.3390/molecules28114312/s1, Figure S1: FT-IR spectrum of the as-prepared Mn_3_O_4_ sample; Figure S2: SEM images of (a1–a3) Mn_3_O_4_/NF-0.12 and (b1–b3) Mn_3_O_4_/NF-0.24; Figure S3: The adsorption of AO7 on Mn_3_O_4_/NF-0.48; Figure S4: AO7 degradation efficiency (a) and kinetic curves (b) of Mn_3_O_4_/NF-0.48/PMS system under different stirring rates. Conditions: [AO7]_0_ = 20 mg/L, [oxone]_0_ = 1 mM; Figure S5: EPR spectra of Mn_3_O_4_/NF-0.48/PMS system captured by TEMP.
