# Supplementary material for: Peroxymonosulfate-Activation-Induced Phase Transition of Mn3O4 Nanospheres on Nickel Foam with Enhanced Catalytic Performance
Source: Molecules. 2023 May 24;28(11):4312. doi: 10.3390/molecules28114312 (PMC10254458; doi:10.3390/molecules28114312)
Supplement: Supplementary file 1 [file molecules-28-04312-s001.zip › molecules-2364256-supplementary.pdf]

Supplementary Information

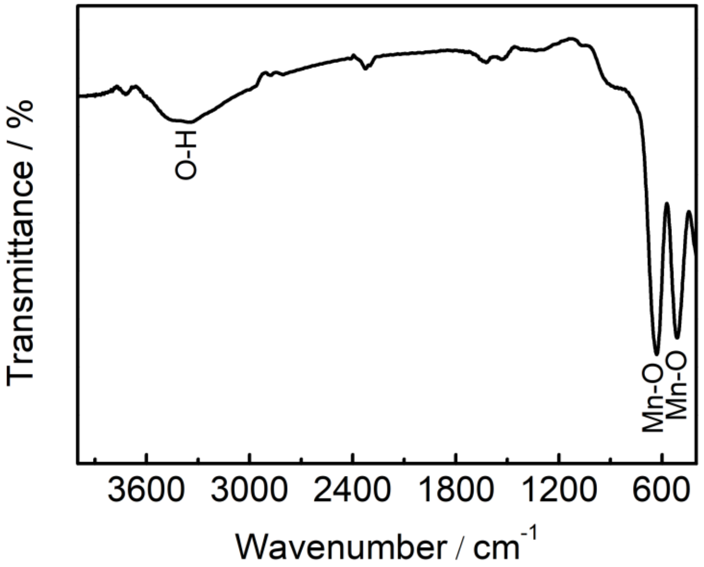

Figure S1. FT-IR spectrum of the as-prepared Mn_3O_4 sample.

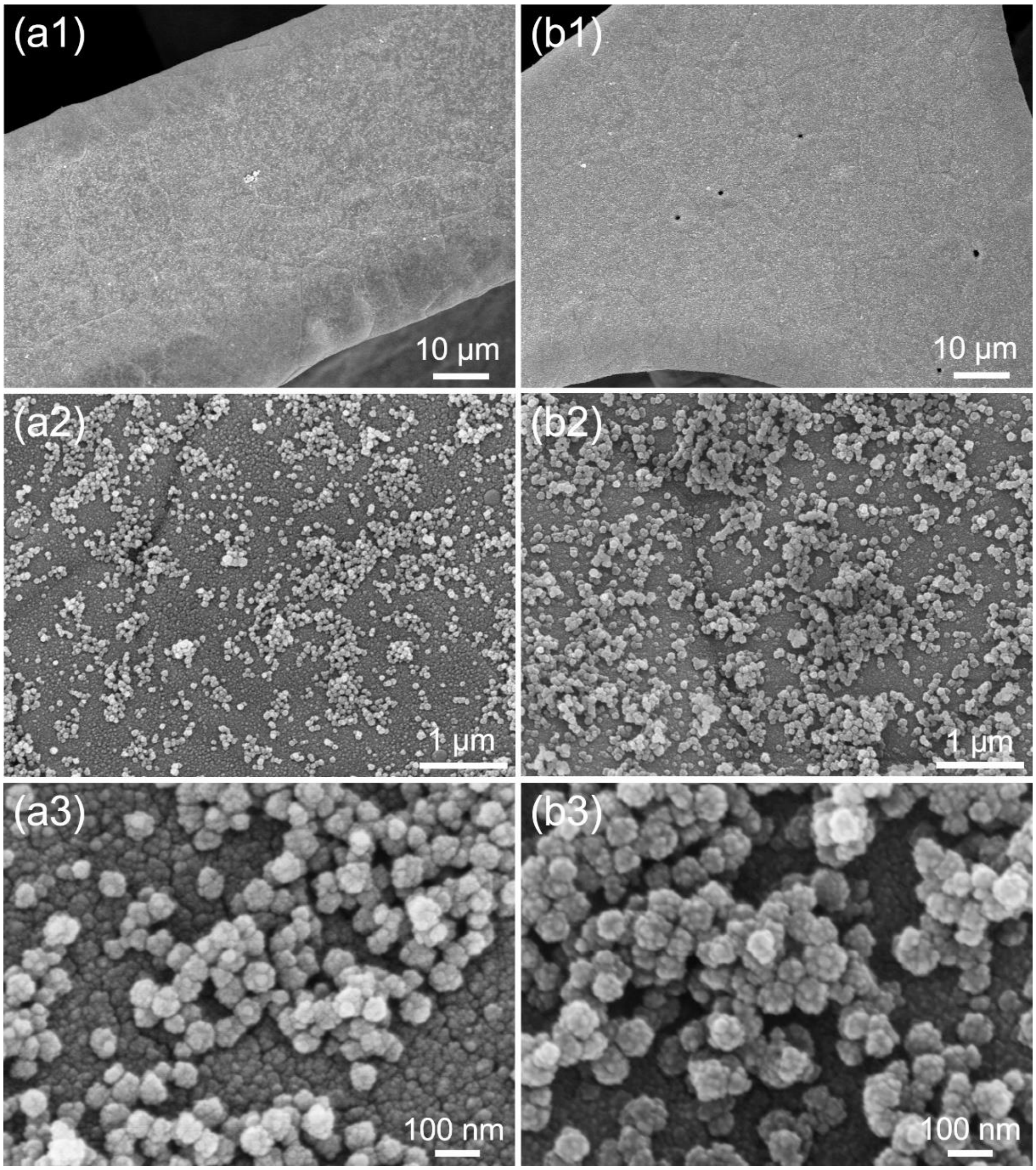

Figure S2. SEM images of (a1-a3) Mn_3O_4/NF-0.12 and (b1-b3) Mn_3O_4/NF-0.24.

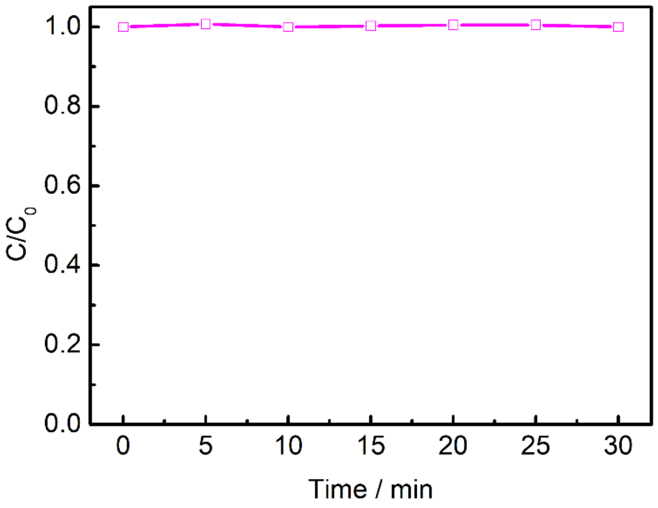

Figure S3. The adsorption of AO7 on Mn_3O_4/NF-0.48.

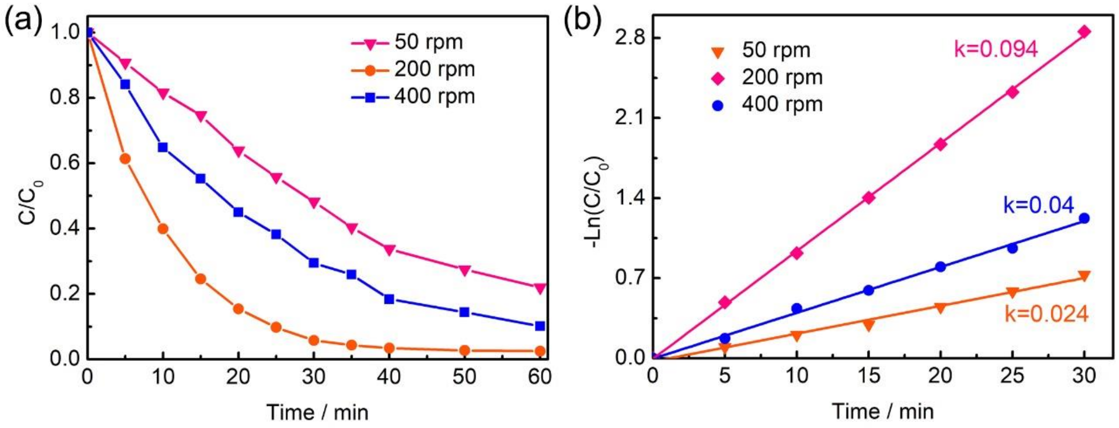

Figure S4. (a) AO7 degradation efficiency and (b) kinetic curves of Mn_3O_4/NF-0.48/PMS system under different stirring rates. Conditions: $[AO7]_0$=20 mg/L, $[oxone]_0$=1 mM.

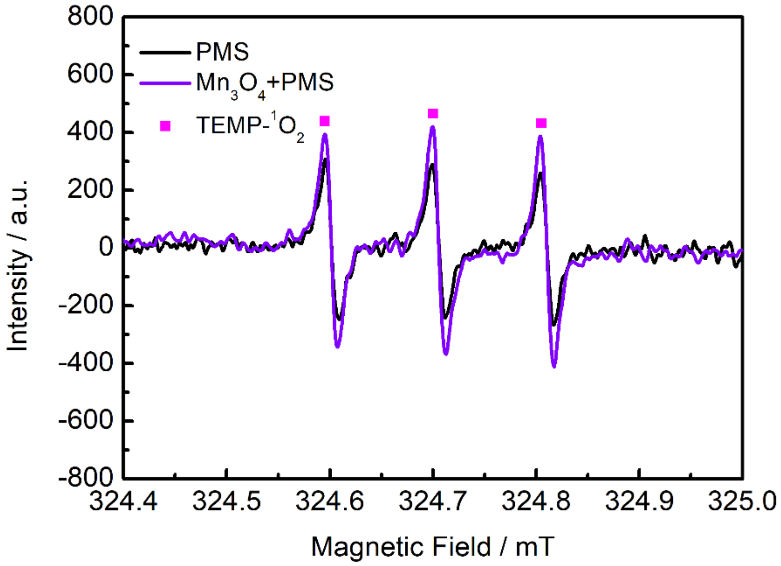

Figure S5. EPR spectra of Mn_3O_4/NF-0.48/PMS system captured by TEMP.
